# Cannabis use in endometriosis: the patients have their say—an online survey for German-speaking countries

**DOI:** 10.1007/s00404-024-07652-6

**Published:** 2024-08-09

**Authors:** Victoria Jasinski, Renata Voltolini Velho, Jalid Sehouli, Sylvia Mechsner

**Affiliations:** Department of Gynecology with Center of Oncological Surgery, Endometriosis Research Center Charité, Campus Virchow-Klinikum, Augustenburger Platz 1, 13353 Berlin, Germany

**Keywords:** Endometriosis, Endocannabinoid system, Cannabis, Pain management, Self-management

## Abstract

**Purpose:**

Endometriosis is a chronic inflammatory disease that can cause various pain symptoms. Current therapy options do not always provide sufficient pain relief and often cause unpleasant side effects. Recent studies have shown that the endocannabinoid system is involved in the endometriosis pathophysiology, and using Cannabinoids may be a potential therapeutic option. We aimed to determine for the first time, the Cannabis use prevalence, self-rated effectiveness, and the possible reduction in medication in German-speaking countries.

**Methods:**

A cross-sectional online survey was distributed through endometriosis support and advocacy groups on social media. German-speaking endometriosis patients aged ≤ 18, residing in Germany, Austria, and Switzerland were eligible to participate.

**Results:**

Out of 912 participants who provided valid answers, 114 reported using cannabis for self-management. Cannabis was rated as the most effective self-management strategy to reduce symptom intensity (self-rated efficacy 7.6 out of 10). Additionally, ~ 90% of the participants were able to decrease their pain medication intake. The greatest improvement was observed in sleep (91%), menstrual pain (90%), and non-cyclic pain (80%). Apart from increased fatigue (17%), side effects were infrequent (≤ 5%).

**Conclusion:**

At the time of the study, Cannabis consumption was still illegal in Germany, Austria, and Switzerland, with medical cannabis being rarely prescribed due to complex requirements. Results suggest that Cannabis has become a popular self-management method for treating endometriosis-related symptoms, leading to substantial symptom improvement. Further studies are needed to investigate the best administration methods, dosage, THC/CBD ratio, potential side effects, and long-term effects to provide official recommendations to patients and healthcare providers.

## What does this study add to the clinical work

**Table:** 

Self-management strategies are commonly used in several parts of Europe, with cannabis being described as the most effective method for managing endometriosis-related symptoms. The study indicates that there is a significant interest and demand for additional therapeutic options, and cannabis can potentially become an important part of a multimodal therapy approach for treating endometriosis.	

## Introduction

Endometriosis is defined as a benign chronic inflammatory disease with endometrial-like tissue outside the cavum uteri, affecting 2–20% of women of reproductive age [1, 2]. Even though it is one of the most common benign gynecological diseases, it takes 4–11 years on average from the beginning of the first symptoms to the diagnosis [3]. The main symptoms are cyclic or acyclic chronic pelvic pain, dysmenorrhea, dyspareunia, dysuria, and dyschezia [4]. Associated with infertility rates of 30–50%, endometriosis extends its influence on pain disorders, irritable bowel syndrome, and depression, amplifying limited quality of life [5, 6]. Current therapy options include hormonal treatments and laparoscopic surgery, but pain recurrence is common [7]. Since chronic pain perception can lead to central sensitization, adequate pain management is of great importance [8]. Pain management involves the use of non-steroidal anti-inflammatory drugs (NSAIDs), metamizole, and in severe cases opioids [9]. However, these therapies often come with notable side effects, underscoring the urgent need for novel treatments that efficiently alleviate symptoms and prevent recurrence while preserving fertility [10]. Additionally, self-management strategies like a balanced diet, regular exercise, the use of heat, as well as physiotherapy are used very often and can help to relax the pelvic muscle [11]. Due to the insufficient data, no universally applicable guidelines regarding lifestyle modifications have been formulated so far. However, some studies indicate that symptoms may improve with an antioxidant and anti-inflammatory diet, increased intake of omega-3 fatty acids, and vitamins C, D, and E, as well as a low-FODMAP diet [12]. As mental health can affect pain perception, psychotherapy or relaxation therapy may also prove beneficial [9].

In a recent systematic review about cannabinoids and endometriosis, covering the period from 1946 to 2021, 53 publications were identified [13]. Among them, only 10 laboratory-based studies in human samples were found, involving approximately 223 endometriosis patients, 165 controls, and 94 non-endometriosis women. Women with endometriosis exhibited decreased expression of cannabinoid receptor 1 (CB1R) in lesions compared to controls, as well as increased levels of the endogenous ligands of CB1R and CB2R, Arachidonoylethanolamine (AEA), and 2-Arachnidonoyl glycerol (2-AG). This suggests a negative feedback loop that may contribute to inflammation [14]. A recent case–control study involving 23 cases and 19 controls found that endocannabinoid levels were significantly influenced by both non-cyclic and cyclic abdominal pain. Women with non-cyclic abdominal pain showed higher 2-AG levels in the peritoneal fluid throughout the menstrual cycle, while women with dysmenorrhea had higher 2-AG levels and lower AEA levels during the proliferative phase alone [15]. Furthermore, 2-AG levels positively correlated with prostaglandin E_2_ (PGE_2_), and the AEA/2-AG ratio positively correlated with defensins, suggesting a potential connection between the endocannabinoid system and inflammatory pain [15].

In vitro studies using human material have indicated that exogenous cannabinoids may correct dysregulations. Treatment of endometrial cells with the cannabinoid agonist WIN 5512–2 resulted in decreased cell proliferation, reduced reactive oxygen species production, and decreased expression of alpha-smooth muscle actin, providing evidence of the anti-inflammatory effects of cannabinoids [16]. Endocannabinoids have also been shown to stimulate endometrial cell migration [17]. Bilgic et al. [18] observed that CB1R and CB2R expressions are decreased in endometriosis tissue compared to control, along with decreased apoptosis indexes. Moreover, exposure of endometriosis tissue to CB1R and CB2R agonists led to pro-apoptotic effects. Considering the role of the endocannabinoid system in endometriosis modulation, cannabinoids have been suggested as a potential therapy.

In summary, the limited and controversial basic research data do not allow or support the final recommendation to use cannabinoids in the treatment of endometriosis. However, cross-sectional surveys with endometriosis patients from Australia, New Zealand, Canada and the USA showed that self-management strategies are very common in those patients and that cannabis and cannabis products are among the most effective at pain reduction [19–21]. Thus, we aimed to determine for the first time, the Cannabis use prevalence, self-rated effectiveness, and the possible reduction in medication in German-speaking countries.

## Methods

### Survey development and design

The research team developed an online questionnaire (Additional File 1) based on two previous surveys conducted in New Zealand and Australia [19, 22]. Self-management was defined as non-pharmacological interventions, such as physical or psychological techniques, that women could perform themselves, or lifestyle interventions, such as dietary changes, exercise, alcohol or cannabis use, that were undertaken specifically for the management of endometriosis symptoms. The questionnaire required participants to recall measures taken in the past 6 months. It collected demographic information, endometriosis-related questions, use of self-management techniques in the previous 6 months, reasons for non-usage of self-management, type, frequency of self-management used, adverse events, self-rated effectiveness, and any reduction in endometriosis-related medication use. This article provides an overall summary of cannabis self-management, while the analysis of all self-management strategies will be published separately.

The survey took approximately 15–45 min to complete. Women aged 18–55, currently living in Germany, Austria, and Switzerland, who spoke German and had a confirmed diagnosis of endometriosis were eligible to participate. Recruitment was conducted via a direct link to the survey, and an invitation to participate was distributed through the social media platforms (Facebook, Twitter, and Instagram) of endometriosis support and advocacy groups in those three countries. The survey link was active from August to December 2022.

For the evaluation of endometriosis-associated pain and the self-rated effectiveness of self-management methods, an 11-point numerical rating scale (NRS) from 0 to 10 was utilized. In this context, 0 indicates “no pain” or “not effective at all” and 10 “strongest pain” or “very effective”. The evaluation of the impact of pelvic pain on different aspects of life was conducted using a 5-point scale, where 1 represents no impact at all and 5 extreme impacts.

### Data analysis

The data were analyzed using IBM SPSS version 29. Mean and standard deviation were used to present normally distributed variables, while median and interquartile range were used for non-normally distributed variables. Categorical variables were reported in absolute numbers and relative percentages. A t-test for independent samples was used to determine the statistical comparison of mean values, while a chi-square test was used for categorical variables. As not all participants answered all questions, the number of responses is listed individually for each variable. Free responses were transformed into Excel, analyzed manually, and sorted into subgroups. Ambiguous responses were discussed to arrive at a mutually acceptable solution.

## Results

Out of 1403 participants who started the survey, 912 individuals completed all the questions and were included in the analysis. Among those, over 75% (*n* = 688) reported trying self-management strategies in the past 6 months to better cope with endometriosis symptoms or reduce the side effects of medications used to treat the condition. Interestingly, 17% (*n* = 114) of the respondents used cannabis or cannabis-related products as a self-management method.

Table 1 outlines the demographic characteristics of the participants. On average, cannabis users were 30 ± 6 years old, and the majority of them were from Germany (96%). Most of the endometriosis patients reported being in a committed relationship or marriage. In comparison to non-cannabis users, cannabis users had lower incomes and were more likely to be unemployed (17% vs. 3%, *p* < 0.001). Further, more than twice as many cannabis users smoke cigarettes compared to non-users (41% vs. 19%, *p* < 0.001).

**Table 1:** Sociodemographic data among cannabis users and non-users

	All (912) Mean (SD) or n (%)	Non-users Mean (SD) or n (%)	Cannabis users Mean (SD) or n (%)	*P*-value
Age (years)	30.6 (6.51)	30.73 (6.54)	29.68 (6.25)	0.427^a^
*n* =	891	780	111	
Country				
Germany	863 (94.9)	755 (94.8)	108 (95.6)	0.890^b^
Austria	31 (3.4)	28 (3.5)	3 (2.79)	
Switzerland	15 (1.7)	13 (1.6)	2 (1.8)	
*n* =	909 (100)	796 (100)	113 (100)	
Highest level of education				
School student	2 (0.2)	0	2 (1.8)	0.023^b^
No graduation	4 (0.4)	4 (2.6)	0	
Lower secondary school graduates	25 (2.7)	21 (2.6)	4 (3.5)	
Secondary school graduates	219 (24.0)	188 (23.6)	31 (27.2)	
Technical college certificate	126 (13.8)	111 (13.9)	15 (13.2)	
Finished high school	204 (22.4)	176 (22.1)	28 (24.6)	
University degree	314 (34.4)	281 (35.1)	33 (28.9)	
Other	18 (2.0)	17 (2.1)	1 (0.9)	
*n* =	912 (100)	798 (100)	114 (100)	
Employment status				
Working	754 (82.8)	679 (85.2)	75 (65.8)	< 0.001^b^
Unemployed	45 (4.9)	26 (3.3)	19 (16.7)	
Retired	18 (2.0)	12 (1.5)	6 (5.3)	
Housewife	21 (2.3)	17 (2.1)	4 (3.5)	
Other	73 (8.0)	63 (7.9)	10 (8.8)	
*n* =	912 (100)	797 (100)	114 (100)	
Net income				
Non	50 (5.5)	33 (4.1)	17 (14.9)	< 0.001^b^
< 500€	52 (5.7)	42 (5.3)	10 (8.8)	
500–1000€	102 (11.2)	81 (10.2)	21 (18.4)	
1000–1500€	143 (15.7)	128 (16.0)	15 (13.2)	
1500–2000€	163 (17.9)	147 (18.4)	16 (14.0)	
2000–2500€	234 (25.7)	218 (27.3)	16 (14.0)	
2500–3000€	91 (10.0)	78 (9.8)	13 (11.4)	
> 3000€	77 (8.4)	71 (8.9)	6 (5.3)	
*n* =	911 (100)	797 (100)	114 (100)	
Marital status (multiple answers)				
Single	176 (18.9)	159 (19.5)	17 (14.8)	< 0.001^b^
Single parent	22 (2.4)	21 (2.6)	1 (0.9)	
Married	264 (28.4)	248 (30.4)	16 (13.9)	
Committed relationship	446 (47.9)	372 (45.6)	74 (64.3)	
Separated	16 (1.7)	12 (1.5)	4 (3.5)	
Other	7 (0.8)	4 (0.5)	3 (2.6)	
*n* =	931 (100)	816 (100)	115 (100)	
Smoking cigarettes	200 (21.9)	153 (19.2)	47 (41.2)	< 0.001^b^
*n* =	912 (100)	798 (100)	114 (100)	

^a^t-test for unpaired samples

^b^Chi-square test

### Endometriosis-related data

On average, it took 9 years (± 6) from the onset of symptoms to receive a diagnosis. Surgery was the most common method of diagnosis (84%). While the tolerance of the currently taken hormone preparation was mostly assessed as “medium” by the participants (60%), the pain levels during this treatment were on average a 6 (± 3) on the NRS. Those who used cannabis provided hormonal treatments a one-point lower effectiveness rating compared to non-users (4 ± 3 vs. 5 ± 3, respectively; *p* = 0.001).

**Fig. 1:**
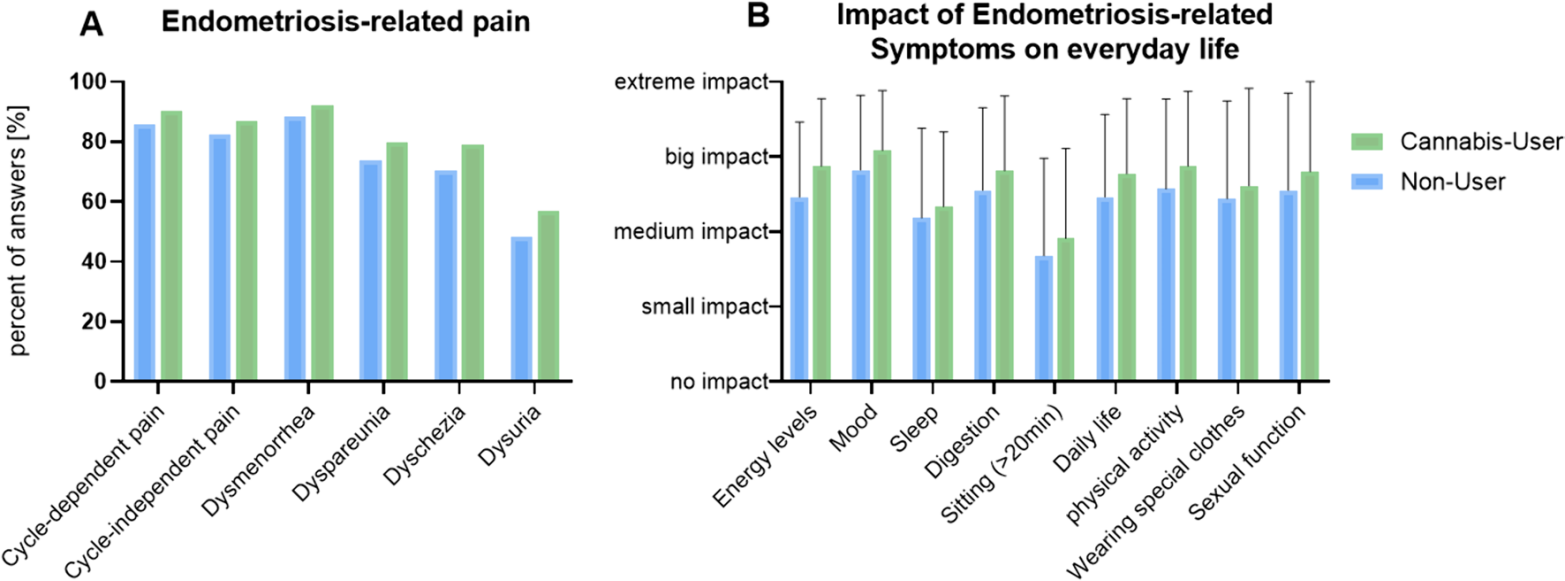
Endometriosis-related data in Cannabis Users and Non-Users. **A** Endometriosis-related pain in cannabis users and non-users. **B** Impact of endometriosis-related symptoms on everyday life of cannabis users and non-users

More than 90% of women experience pain related to their menstrual cycle, while 87% experience non-cycle-related pain. Pain during defecation and sexual intercourse was reported by almost 80% of women (Fig. 1a). Endometriosis caused significant restrictions in various aspects of women’s lives, including mood, performance, digestion, sexual behavior, physical activity, daily living, and clothing choices, with around 80% of women feeling "extremely" to “highly” restricted. Among cannabis users, women felt more limited than non-users in all areas (Fig. 1b). The extent to which endometriosis influences the relationship was measured on a scale from 0 (no influence) to 5 (substantial effect) due to factors like pain, libido changes, or fertility issues. The results showed that among cannabis users, the influence was rated at 3.4, and among non-users, it was rated at 3.1 out of 5 (*p* = 0.024).

Individuals who use cannabis were more likely to have tried different pain medications in the past (see Additional File 2). They also reported taking opioids, such as tramadol (*p* < 0.001) and tilidine (*p* = 0.001), at about twice the rate of non-users for pain relief. Under the influence of analgesic medication, cannabis users rated their endometriosis-related pain intensity at an average of 6 (± 2) on the NRS. The effectiveness of pain reduction was assessed at an average of 4 (± 2) with 10 representing a maximum of effectiveness. On the other hand, non-users reported pain intensity and effectiveness of 5 (± 2), indicating that the group of cannabis users experienced more severe pain (*p* < 0.001) and perceived pain medication to be less effective (*p* = 0.001).

### Cannabis-related data

114 individuals (17%) reported using cannabis to manage their endometriosis-related symptoms in recent months. The frequency and the method of cannabis use varied greatly among the users. Smoking was the most commonly utilized method (*n* = 106) and was used more often, several times daily (*n* = 27), or several times a week (*n* = 23), compared to other methods. Consumption through vaporization (*n* = 69), as edibles (*n* = 72), or other methods (*n* = 75) was generally less frequent, typically once a week. The median cost for cannabis is 50 Euros per month, with variations ranging from zero expenses for some users to a maximum of 550 Euros for others. The highest enhancement in symptom relief with cannabis use was observed in the areas of sleep (91%), menstrual pain (90%), and non-cyclical pelvic pain (80%). However, cannabis appears to be less effective in reducing pain during sexual intercourse, bowel movements, and urination.

**Fig. 2:**
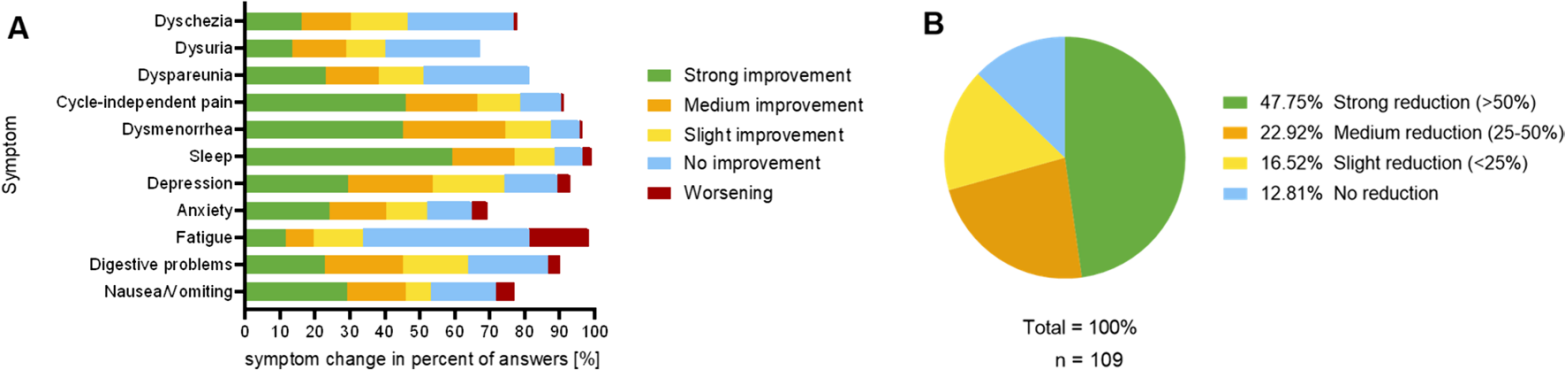
Self-reported change in endometriosis-related symptoms and reduction in pain medication due to Cannabis intake. **A** Self-reported change in endometriosis-related symptoms due to Cannabis intake. **B** Reduction in Pain-medication due to Cannabis intake

Cannabis also showed potential in alleviating psychological symptoms, as reported by three-quarters of women with anxiety or depression/depressed mood who experienced improvement. However, a small number of users (> 5%) reported a worsening of these psychological complaints. The most common side effect was increased fatigue (17%), followed by increased nausea and vomiting (5%). Apart from fatigue, these other side effects often occur in combination. The exact distribution of the effects and side effects can be seen in Fig. 2a. On average, pain intensity with cannabis use was rated at 4.2 (± 2.7), with effectiveness in reducing endometriosis-related pain rated at 7.6 out of 10 (± 2.6).

Figure 2b shows that nearly 90% of respondents were able to use cannabis to reduce the amount of pain medication needed, with nearly half of them achieving a strong reduction (> 50%). Ultimately, almost 90% of respondents would recommend cannabis as an option for managing endometriosis-associated symptoms.

In the free-text responses, individuals have exemplified their expectations regarding cannabis consumption, stating, for instance, “My expectations were exceeded. I am pain-free and have regained my quality of life!” or “I was initially negative about the whole thing because I thought it was a harmful drug, but it helps like a miracle.” Many women report that cannabis is sometimes the only or most effective treatment for endometriosis-related pain. They note its positive impact on accompanying symptoms, such as nausea, reduced appetite, and sleep problems: “The pain-induced nausea and dizziness have almost disappeared through consumption. I enjoy eating again.”

Regarding the side effects of cannabis, some individuals find the occurrence of fatigue pleasant, leading to fewer sleep problems in the evening and at night. However, for others, this fatigue becomes a significant disadvantage, primarily limiting its use in the morning. It becomes evident that experiences related to the psychological effects of cannabis vary widely. While some users report “reduced anxiety/despair” and improvements in mental health, others have observed a worsening of these conditions. However, almost all responses focused on structural issues: cannabis is challenging to obtain, physicians are poorly or not informed at all, cost coverage by health insurance is laborious and partially unsuccessful, dosages vary significantly, and there are few alternatives in terms of administration methods. Additionally, concerns exist regarding stigmatization in the workplace and personal environment, impaired driving ability, and the potential for dependency.

### Interest in Cannabinoid-based medical products as a treatment option for endometriosis-related symptoms

More than 80% (*n* = 734) of all respondents expressed interest in using a CBD-based product, while more than 60% (*n* = 551) were open to using a THC-based product to treat endometriosis-related symptoms. This proportion increased even further for both substances (87% for CBD and 70% for THC) when prescribed by a physician. The preferred mode of administration for both substances was oral/sublingual (87% for CBD and 71% for THC), followed by topical forms, such as creams (68% for CBD and 56% for THC) and patches (64% for CBD and 55% for THC). However, a relatively small percentage of participants were willing to use CBD- or THC-containing agents vaginally, for example, through suppositories (40% for CBD and 33% for THC).

## Discussion

Our research provides, for the first time, a comprehensive overview of the use of Cannabis and cannabis-based products as a therapeutic option in German-speaking endometriosis patients. We surveyed 912 participants, revealing that over 75% of them tried self-management strategies in the past 6 months to cope with endometriosis symptoms or reduce the side effects of medications. Of these, 114 (17%) used cannabis or cannabis-related products as a self-management method. The use of Cannabis had a significant impact on the overall well-being and quality of life of women with endometriosis. Although cannabis was used comparatively infrequently, it was rated the most effective of all self-management strategies. Noticeable improvements in symptoms were particularly evident in the domains of sleep, chronic pelvic pain, and dysmenorrhea. Furthermore, nearly nine out of ten women were able to reduce the amount of pain medication they consume.

Our results closely align with the work of Sinclair et al. [22] conducted in Australia, with 484 complete answers and 48 cannabis users. The proportion of use of self-management strategies is similar, but the percentage of cannabis users in our study is slightly higher (13% vs. 17%). In Canada, as a country that introduced the legalization of cannabis in 2018, prevalent use of 54% was determined among endometriosis patients [23]. Both studies indicate that individuals who use cannabis have higher scores in terms of the impact of pelvic pain on their daily lives, compared to those who do not use. This suggests that the limitations caused by endometriosis are greater for cannabis users. The effectiveness of pain reduction was estimated on a scale of 0–10, with a range of 7.6 to 8 for both studies. The results of the survey showed differences in the reduction of pain medication between the German-speaking countries and Australia. The Australian survey reported more positive results, which could be due to variations in the use of analgesics or dosages for chronic pain conditions in Australia. Moreover, the study found that daily cannabis use was less common in German-speaking countries compared to Australia. This suggests that the Sinclair et al.’s study may have had a more consistent effect of cannabis, leading women to either reduce or completely forgo additional analgesic intake to a greater extent.

Even though the first available data on the use of cannabis in endometriosis suggest that the substance can have a positive effect on the patient’s complaints, the prescription of the substance is a major barrier for many treating physicians in German-speaking countries. The prescription is only possible if there is presence of a severe disease for which other recognized treatment options are either not available or not possible. In the case of endometriosis, any hormone and pain therapies, including the use of opiates, would have to be insufficiently effective before an application for a prescription of cannabinoids could be made. In addition, there must be a prospective improvement in symptoms resulting from the cannabinoids used. Notably, physicians, especially gynecologists in German-speaking countries, lack experience in prescribing Cannabinoids. While some rare pain specialists work with Cannabinoid-Based Medical Products, they are not specialized in endometriosis. The special pain character of endometriosis patients, involving both cyclical and non-cyclical pain and a wide range of symptoms, necessitates highly trained physicians for pain management AND endometriosis. Patients with chronic pelvic pain and endometriosis face limited multimodal treatment options covered by the health system, with only hormones, surgeries, and painkillers available. This deficiency in the standard care leaves patients feeling helpless, prompting them to seek self-management strategies. Since the 1st April 2024, the consumption of non-medically prescribed cannabinoids is legal in Germany [24]. This allows patients who have been using this drug illegally as a self-management strategy to have legal access to cannabis of high quality, and thus better control the amount of total CBD and THC content. Nevertheless, it is important to note that this cannot serve as a substitute for a proper medical consultation that guides the appropriate dosage and usage instructions, along with information on potential risks and side effects. Such a situation is not desirable from a medical, political, or patient perspective.

In Austria, although the prescription of medical marijuana extract is legal, there are no plans to change the law regarding the legalization of non-medical cannabis for the time being [25, 26]. Switzerland, on the other hand, has started the legal sale of cannabis through pilot projects; outside of these projects, cannabis-containing products with a THC content of less than 1%, as well as medically prescribed cannabis, are legal [27].

It has been shown by various research groups that Cannabinoids can have positive effects on patients with endometriosis. However, to verify these findings, clinical trials with a defined dose, application form, and frequency are required. It is important to focus not only on symptom improvement but also on side effects. Since most endometriosis patients are young women of childbearing age, it is critical to thoroughly investigate other possible consequences, such as the development or intensification of psychoses or influences on the embryo in case of pregnancy. Considering the relatively young age of users, particularly with over 50% of Cannabis Users under 30, it is crucial to prioritize examining the potential adverse effects of cannabinoids on mental health and cognitive development as a side effect.

Our study had several strengths. First, it represents the first work on cannabis use among endometriosis patients in Europe. Second, it has a large number of participants with over 900 valid responses. Third, good comparability to the data from Australia could be achieved using a similar questionnaire. However, there are also limitations. First, it should be emphasized that only subjectively assessed parameters were asked in this survey, which means that no objective effectiveness of cannabis in endometriosis can be assessed. Second, by sharing the survey via digital channels, it cannot be excluded that mainly women who feel more restricted and burdened by their disease participate. Third, this work cannot provide more specific results regarding the mode of application, frequency, or dosage of cannabis and cannabis products. Another study has shown that depending on the corresponding symptom, certain forms of application were perceived to improve symptoms to varying degrees [28].

This study highlights that self-management strategies are commonly used in several parts of Europe, with cannabis being described as the most effective method for managing endometriosis-related symptoms. Cannabis use resulted in a significant improvement in symptoms, going beyond just pain management, and a majority of users were able to reduce their pain medication intake. Adverse effects were found to be rare. Nevertheless, more research is required to determine the best route of administration, dosage, THC/CBD ratio, potential side effects, and long-term effects of cannabis use. This will help provide official recommendations to patients and healthcare providers. The study indicates that there is a significant interest and demand for additional therapeutic options, and cannabis can potentially become an important part of a multimodal therapy approach for treating endometriosis.

## Data Availability

Data is available upon request.
